# Technologies to Study Action Potential Propagation With a Focus on HD-MEAs

**DOI:** 10.3389/fncel.2019.00159

**Published:** 2019-04-26

**Authors:** Vishalini Emmenegger, Marie Engelene J. Obien, Felix Franke, Andreas Hierlemann

**Affiliations:** ^1^Department of Biosystems Science and Engineering, ETH Zürich, Basel, Switzerland; ^2^MaxWell Biosystems AG, Basel, Switzerland

**Keywords:** axon, action potential propagation, patch-clamp technique, genetically encoded voltage indicators, high-density microelectrode arrays

## Abstract

Axons convey information in neuronal circuits via reliable conduction of action potentials (APs) from the axon initial segment (AIS) to the presynaptic terminals. Recent experimental findings increasingly evidence that the axonal function is not limited to the simple transmission of APs. Advances in subcellular-resolution recording techniques have shown that axons display activity-dependent modulation in spike shape and conduction velocity, which influence synaptic strength and latency. We briefly review here, how recent methodological developments facilitate the understanding of the axon physiology. We included the three most common methods, i.e., genetically encoded voltage imaging (GEVI), subcellular patch-clamp and high-density microelectrode arrays (HD-MEAs). We then describe the potential of using HD-MEAs in studying axonal physiology in more detail. Due to their robustness, amenability to high-throughput and high spatiotemporal resolution, HD-MEAs can provide a direct functional electrical readout of single cells and cellular ensembles at subcellular resolution. HD-MEAs can, therefore, be employed in investigating axonal pathologies, the effects of large-scale genomic interventions (e.g., with RNAi or CRISPR) or in compound screenings. A combination of extracellular microelectrode arrays (MEAs), intracellular microelectrodes and optical imaging may potentially reveal yet unexplored repertoires of axonal functions.

## Introduction

Intricate operations, performed by neuronal networks, emerge from the orchestrated interplay of individual neurons. Neurons use action potentials (APs) as a means to encode and relay information from the soma to the presynaptic terminal via reliable conduction through the axon. The three functional compartments of the axon include the axon initial segment (AIS), the axon proper, and the presynaptic terminal. Somato-dendritic integration of a number of synaptic inputs at the AIS are thought to shape the AP firing patterns. The axon proper is often conceived as a simple cable, whose function is the faithful transmission of the AP to distant presynaptic terminals in a digital (all or none) fashion. However, with the development of modern techniques that can directly access small axonal structures, an increasing body of work has emerged that challenges the traditional view on the role of the axon being purely limited to the transmission of the AP(Debanne et al., 2011; Sasaki et al., 2011; Sasaki, 2013; Bucher, 2016). It has been shown that the shape of the presynaptic AP can be modulated by subthreshold potentials, which, in turn, modulate the spike-evoked transmission through so-called “analog-digital facilitation” (Debanne, 2004; Alle and Geiger, 2008; Kress and Mennerick, 2009; Bucher and Goaillard, 2011; Debanne et al., 2011; Sasaki, 2013; Bucher, 2016). As AP propagation and synaptic transmission might undergo substantial modulation, the computational repertoires of single axons in the neuronal circuit may be more complex than commonly assumed.

Axonal membrane excitability and conduction velocity can change substantially with repeated activation. This can potentially alter the temporal patterns of spikes during propagation from the AIS to presynaptic sites. Such changes in temporal spike patterns may be an important feature of neural coding strategies (Izhikevich, 2006; Bucher and Goaillard, 2011; Bucher, 2016). Axonal conduction velocity in unmyelinated axons depends on several biophysical factors, such as ion-channel densities and kinetics, membrane capacitance, axial resistance, axon geometry, and, for myelinated axons, myelin thickness and internodal distances (Hodgkin, 1954; Manor et al., 1991; Shepherd and Harris, 1998; Ganguly et al., 2000; Fields, 2005; Cai et al., 2011). Axonal conduction velocity *per se* provides little information about the functional aspects of neuronal communication. On the other hand, axonal conduction delay, which depends on both, conduction velocity and axonal length, may have important functional implications in the integration of sensory information (Konishi, 2003). A plethora of diverse neurological disorders is associated with impaired axonal functionality (Suter and Scherer, 2003; Waxman, 2006; Krarup and Moldovan, 2009; Kullmann, 2010; Egawa et al., 2017; Khalilpour et al., 2017). Axonal dysfunction can be caused by missing or reduced myelination (e.g., multiple sclerosis) (Steinman, 1996; Trapp et al., 1999). Acute axonal damage (e.g., traumatic injury) (Smith and Meaney, 2000; Johnson V.E. et al., 2013), toxic entities, aggregated proteins, microgliosis and disrupted axonal transport (e.g., prion disease, Parkinson’s disease, Alzheimer’s disease) (Liberski and Budka, 1999; Millecamps and Julien, 2013) may directly affect axonal physiology. Lastly, abnormalities in the composition or function of ion channels (in channelopathies, e.g., in certain forms of migraine and epilepsy) are known to alter the conduction properties of axons (Goadsby et al., 2017; Oyrer et al., 2018; Pietrobon, 2018).

**FIGURE 1:**
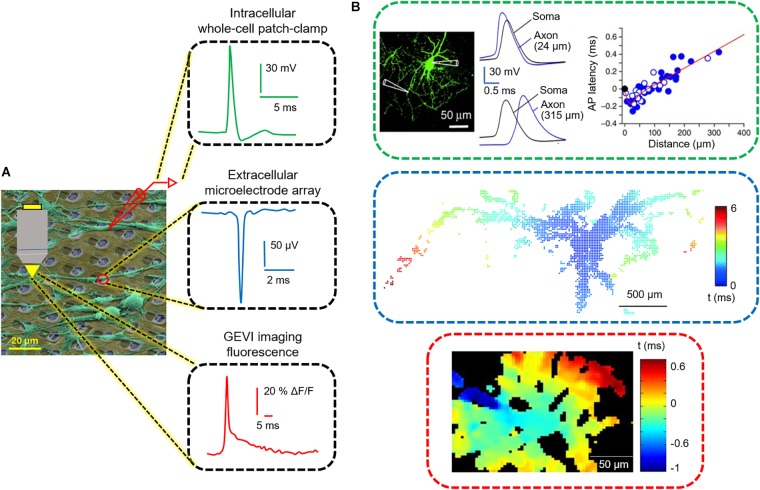
Three functional readouts for measuring neuronal and axonal activity. **(A)** Colored scanning electron micrograph of neurons (green) cultured on a CMOS HD-MEA chip. The readouts include, from top to bottom, patch pipettes, microelectrodes and fluorescence microscopy. At the right side, an intracellular action potential (AP), recorded by using the patch-clamp technique, an extracellular AP recorded by an HD-MEA, and an AP-induced fluorescence signal using GEVIs are displayed. **(B)** Tracking of AP propagation in neuronal processes performed by the three techniques displayed in **(A)**. Modified with permissions from Hochbaum et al., 2014; Hu and Jonas, 2014; Müller et al., 2015.

Recent advances in the understanding of axon physiology and pathophysiology have been driven by technological developments, such as optical imaging of the membrane potentials using genetically encoded voltage indicators (GEVI), subcellular patch-clamp recordings from thin axons and boutons, and high-density microelectrode array recordings (HD-MEA) (Figure 1; Shu et al., 2006; Kole et al., 2007; Sasaki et al., 2011; Bakkum et al., 2013; Novak et al., 2013; Hoppa et al., 2014; Kawaguchi and Sakaba, 2015; Müller et al., 2015; Rama et al., 2015a; Radivojevic et al., 2017). These techniques stand apart from other classical electrophysiological methods in their ability to monitor and interactively control subcellular components of single neurons at high spatial and microsecond temporal resolution. The above-mentioned techniques and devices were reviewed in-depth elsewhere (Hierlemann et al., 2011; Sasaki, 2013; Spira and Hai, 2013; Obien et al., 2015; Inagaki and Nagai, 2016; Ohura and Kamiya, 2016; Yang and St-Pierre, 2016; Xu et al., 2017; Zeck et al., 2017; Platisa and Pieribone, 2018; Rama et al., 2018; Wang et al., 2019). Here, we will briefly introduce these technologies with the focus on studying axonal signals, while we describe - in a bit more detail - recent investigations in axonal neurobiology by using HD-MEAs.

## Technological Approaches

In the following, we provide a brief overview of the three most commonly used recording modalities for measuring axonal signals. We describe the parameters that govern to the detection of neuronal signals and outline recent advances, including potential advantages and limitations. Table 1 lists the key specifications of each methodology. Since several publications are available on each technology, we pooled the data from the most advanced and most recent publications. Given the plethora of applications for each methodology, we will restrict our comparison to the detection and measurements of AP propagation. A schematic overview on the different methodologies along with representative signals is displayed in Figure 1.

**Table 1:** Comparison of the three techniques in studies that showed AP propagation.

	Genetically Encoded Voltage Indicators	Subcellular Patch-clamp	CMOS HD-MEA
Recording type	Optical	Electrical	Electrical
Modality	Fluorescence	Intracellular/ Extracellular	Extracellular
Signal	ΔF/F	μV, mV, pA	μV
Spatial resolution within detection area	3.25 μm in 1.2 × 3.3 mm^2^	NA	17.5 μm pixel in 3.85 × 2.10 mm^2^
Temporal resolution	0.37–1.2 ms	μs	μs
Dynamic range	12–90% ΔF/F per 100 mV	± 1 V	±3 μV to ± 1.6 mV ^c^
		1 to 200 nA ^a^, 0.1 to 20 nA ^b^	
Device noise	2.2 mV at 300 W/cm^2^ 1.5 mV at 800 W/cm^2^	∼40 μV_RMS_ 3.0 pA_RMS_^a^_,_ 1.4 pA_RMS_^b^	2.4 μV_RMS_ (AP band)
Duration of stable recording	Minutes	Hours	>Months
Simultaneous recording sites	Medium; dozens	Low; two sites	High; Thousands
Stimulation capability	No – Needs external stimulation	Yes - Electrical	Yes - Electrical
Advantages	Non-invasive	Single-spike resolution	Non-invasive
	Cell-specific labeling	Precise spike shape	Long-term recording
		tracking	Single-trial APs
		Subthreshold and PSP detection	High throughput
Limitations	Photobleaching	Invasive	Cannot detect subthreshold signals
	Phototoxicity	Time consuming	
	Multiple-trial APs	Low throughput	
		Labor intensive	
References	Hochbaum et al., 2014; Gong et al., 2015	Sasaki et al., 2011; Hu and Jonas, 2014	Müller et al., 2015; Radivojevic et al., 2017

*For subcellular patch-clamp, the device specifications of the Multiclamp 700 b have been taken. Feedback resistors: ^a^ 50 MΩ, ^b^ 500 MΩ. ^c^With a gain of 1024; up to 1 V with reduced gain.*

### Voltage Imaging

In order to monitor neural activity at single-cell resolution, optical methods, such as voltage-sensitive dyes and GEVI, make use of fluorescence signals to detect alterations in voltage (Peterka et al., 2011; Storace et al., 2016). Voltage-sensitive dyes have provided important insights into neuronal electrical signaling ranging from individual neurons to population dynamics (Grinvald et al., 1981; Gross and Loew, 1989; Petersen et al., 2003; Miller et al., 2012; Popovic et al., 2015). Yet, major limitations of voltage-sensitive dyes include cell toxicity, phototoxicity, indiscriminate neuronal and glial staining, and small signal-to-noise ratio (SNR) (Knöpfel et al., 2006; Mennerick et al., 2010).

In the last two decades, considerable efforts have been made to overcome these limitations, which have led to the development of GEVIs. The three main molecular designs of GEVIs – inserted into the plasma membrane – are (1) the fusion of fluorescent proteins (FP) to voltage-sensing domains, (2) the use of opsins, and (3) hybrid opsin-FP pairs (rhodopsin-FRET sensor) (Boyden et al., 2005; Kralj et al., 2011; Akemann et al., 2012; Jin et al., 2012; Tsutsui et al., 2013; Hochbaum et al., 2014; St-Pierre et al., 2014; Gong et al., 2015). Voltage sensitivity (dynamic range, %) is an important parameter of fluorescence indicators, expressed as ΔF/F per 100 mV (−70 to 30 mV), which represents linear changes in fluorescence in response to voltage fluctuations. To detect neuronal activity with high SNR, a combination of key features of voltage indicators, such as bright fluorescence, fast kinetics (rapid response to changes in voltage), large dynamic range, photostability, and efficient plasma membrane localization is desired (Lee et al., 2017; Xu et al., 2017; Piatkevich et al., 2018; Wang et al., 2019).

While there is constant development of new constructs, a recent work by Bando et al. (2019) provided a temporal snapshot of state-of-the-art GEVIs and compared their performances. The authors report that QuasAr2, a rhodopsin-based GEVI, outperforms other GEVIs concerning the optical detection of single APs of neurons *in vitro*, featuring high signal amplitude, fast kinetics and high SNR. In addition, Ace-2N-4AA-mNeon, a rhodopsin-FRET sensor, was shown to resolve individual spikes in single trials without averaging, while suffering from fast photobleaching.

A combination of a red-light-excited QuasAr2 with a spectrally compatible blue-light-activated channelrhodopsin (CheRiff) was co-expressed in neurons via a vector called “Optopatch,” which was targeted at enabling simultaneous all-optical electrophysiology in neuronal cultures or organotypic brain slice cultures (Hochbaum et al., 2014; Kiskinis et al., 2018). The use of this construct enabled mapping of the dynamics of AP initiation and propagation across dendritic and axonal structures at high spatiotemporal resolution (Figure 1). However, significant multi-trial averaging (200–17,000 trials) was required for attaining good enough signal-to-noise characteristics (Hochbaum et al., 2014).

### Subcellular Patch-Clamp Recordings

Patch-clamping is the gold standard technique for studying electrical properties of neurons at unprecedented resolution. The patch-clamp technique uses a glass micropipette that presses against the cell membrane to form a tight gigaohm seal resistance between the cell membrane and the rim of the glass micropipette. In the original cell-attached configuration, activity of single ion channels in the tiny patch of membrane surrounded by the tip of the pipette can be studied. If the patch of membrane under the pipette tip is ruptured by applying pressure, the electrode accesses the inside of the cell in the so-called whole-cell configuration, where the trans-membrane voltage and currents can be directly recorded (Neher and Sakmann, 1976; Ogden and Stanfield, 1994).

Most patch-clamp studies have been conducted on the soma, which is the largest compartment of a neuron (8–30 μm in diameter). One of the limitations of the conventional patch-clamp technique is that the studies on axons encountered technical difficulties due to the thin axonal structure (∼200 nm in diameter). Accordingly, axonal recordings have been mostly restricted to giant axon terminals, such as hippocampal mossy fiber boutons (Geiger and Jonas, 2000; Bischofberger et al., 2006; Boudkkazi et al., 2011) and the Calyx of Held (Forsythe, 1994; Borst et al., 1995; Awatramani et al., 2005). Recordings from thin axons have been obtained from axonal blebs (3–6 μm), which are resealed swellings at the cut ends of axons after brain slicing procedures (Shu et al., 2006, 2007; Kole et al., 2007; Kim, 2014; Rama et al., 2015a). Recently, recordings from intact thin axons have been made possible using a fluorescence-guided patch-clamp technique (Ishikawa et al., 2010; Sasaki et al., 2011, 2012; Hu and Jonas, 2014; Kawaguchi and Sakaba, 2015). Cell-attached extracellular recordings of APs in intact unmyelinated axons (∼1 μm in diameter) have been made using pipettes coated with fluorescently conjugated albumin. However, stable recording was possible for only less than 60 min with ∼50% success rate. Simultaneous whole-cell recordings have been performed from the soma and axon shaft of hippocampal basket cells in acute slices (Hu and Jonas, 2014) as well as in the presynaptic terminals in cerebellar Purkinje cells in cultures (Kawaguchi and Sakaba, 2015) to examine the fidelity of AP propagation.

In recent years, several studies were conducted using paired recordings from two distinct sites along a single axon or from a presynaptic axon terminal and a postsynaptic neuron. These experiments have made considerable contributions to understanding the mechanism of analog-digital facilitation, compartmentalized distribution of ion channels and gating properties, as well as the modulation of short- and long-term synaptic plasticity (Engel and Jonas, 2005; Alle and Geiger, 2008; Sasaki et al., 2011; Hu and Jonas, 2014; Kawaguchi and Sakaba, 2015; Rama et al., 2015b; Rowan et al., 2016). However, due to the limitation in simultaneously recording from multiple sites along the axon, the patch-clamp technique is not capable of tracking the modulation of AP propagation throughout the axon proper.

### CMOS HD-MEAs

The electrical activity of neurons can also be detected extracellularly by means of metal electrodes, arranged in large arrays and known as MEAs. Microelectrodes can record changes in the electric field generated by the moving ions in the extracellular space during the electrical activity of a nearby neuron (Buzsáki et al., 2012; Anastassiou et al., 2013). During an AP, the fast Na^+^ current flows away from the electrode into the cell and results in a negative peak in the extracellular action potential (EAP). Thereafter, a slower current of K^+^ ions flows out of the cell toward the electrode resulting in a positive peak. Most axonal signals show a stereotypical positive-first, triphasic shape. The first, small amplitude positive peak corresponds to a capacitive current, the large negative peak to the Na^+^ current, and the final positive peak to the K^+^ current (Gold et al., 2006). In general, EAPs show heterogeneity in signal shapes and amplitudes depending on the magnitude, polarity, and the distance from the recording site (Nunez and Srinivasan, 2006). In addition, the relative positioning of cells with respect to the location of electrodes has a strong influence on the amplitude of the EAP (Gold et al., 2006). EAPs signal amplitudes are in the range of μV and are usually around three orders of magnitude lower than intracellularly measured signals (mV) (Buzsáki et al., 2012).

Commercially available standard MEAs are an established technology for investigating neuronal network activity. However, they do not allow for targeting individual neurons in a network due to the limited number of electrodes (<300), arranged at a comparably large pitch (>30 μm) (Gross et al., 1993; Jimbo and Robinson, 2000; Stett et al., 2003; Rutten et al., 2007). In order to investigate the properties of individual neurons, CMOS (complementary metal oxide semiconductor)-technology-based, planar, HD-MEAs can be used that enable simultaneous recording from a large number of sites at high spatiotemporal resolution (Eversmann et al., 2003; Berdondini et al., 2009; Frey et al., 2009; Huys et al., 2012; Johnson B. et al., 2013; Bertotti et al., 2014; Jackel et al., 2017; Ogi et al., 2017; Tsai et al., 2017). In contrast to a full-frame readout that is also used with CMOS cameras, our lab has developed a flexible readout approach, where a matrix of switches below the electrodes (total number: 26,000–59,000 electrodes) routes arbitrarily selectable subsets of 1024 or 2048 electrodes to a high-end readout circuitry placed outside the electrode array. This flexible readout approach enables (i) high spatial resolution with electrode densities of 3,000 to 5,000 electrodes per mm^2^ at (ii) good signal quality. However, not all the electrodes can be simultaneously read out, but only subsets in a sequential approach [for technical details, cf. (Ballini et al., 2014; Müller et al., 2015; Dragas et al., 2017; Viswam et al., 2018; Yuan et al., 2018)].

The main advantage of the HD-MEAs is the high spatiotemporal resolution, which allows for detection of signals from thin axons (∼200 nm diameter) and the ability to record APs at microsecond temporal resolution. This degree of resolution helps to efficiently assign detected extracellular spikes to units or neurons through spike sorting (Einevoll et al., 2012; Diggelmann et al., 2018). HD-MEAs enable, owing to the high spatial resolution and non-invasive detection of EAPs, the simultaneous recording at EAPs at hundreds of sites simultaneously along the axonal arbor for up to several days (Bakkum et al., 2013; Radivojevic et al., 2017). CMOS-based HD-MEAs have been used with many *in vitro* preparations, such as dissociated cell cultures (Maccione et al., 2012; Bakkum et al., 2013; Yada et al., 2016; Amin et al., 2017; Radivojevic et al., 2017), cultures of induced pluripotent stem cells (iPSCs) (Amin et al., 2016; Fiscella et al., 2018), acute retinae (Menzler and Zeck, 2011; Fiscella et al., 2012; Jones et al., 2015), acute brain slices (Egert et al., 2002; Frey et al., 2009; Ferrea et al., 2012; Medrihan et al., 2014; Obien et al., 2019), and organotypic brain slice cultures (Gong et al., 2016). A disadvantage of HD-MEAs is that inferences with respect to analog signaling are difficult, as the subthreshold signals are not directly measurable.

## Studies of Axonal Neurobiology Using HD-MEAs

**FIGURE 2:**
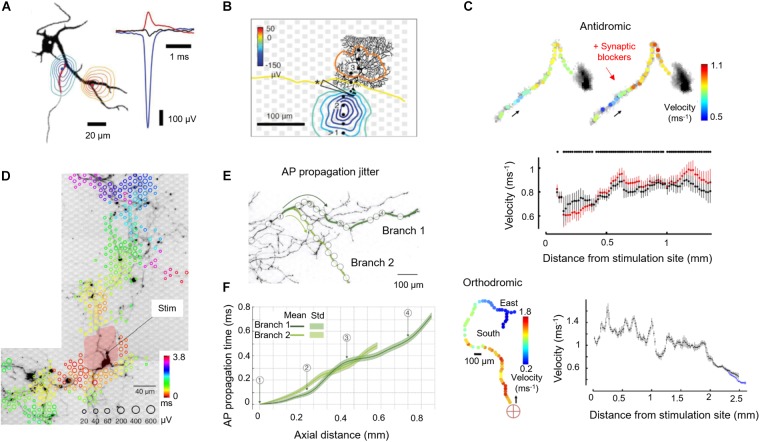
Application of HD-MEAs to study axon neurobiology. **(A) (Left)** Contour plots of the minimum (blue) or maximum (red) extracellular-action-potential (EAP) signal within ± 500 μs of the negative peak. The AIS has been marked on the MAP2 fluorescence image in the background (black) with a red line (ankyrin-G staining). The contours have been normalized to the largest negative signal (blue-to-green) or the largest positive signal (red-to-yellow), see right panel. **(Right)** The largest negative (blue) and positive EAP signals (red) along with the somatic potentials (black) are shown as peaks. **(B)** Spatial distribution of the averaged spontaneous EAP of a Purkinje cell (PC). The largest EAP amplitudes were found along the axon of the PC. **(C) (Top)** Velocity profiles (color) along the propagation pathway without (left) and with (right) application of synaptic blockers. Arrows indicate the antidromic propagation direction. **(Middle)** Velocities without (black) and with (red) application of synaptic blockers calculated by using a bootstrapping procedure. **(Bottom)** The same analysis was performed for an orthodromic action potential. The red cross indicates the stimulation electrode located near the soma. Propagation continued into two branches (“East” and “South”). **(D)** Stimulation-triggered EAP footprint superimposed with neuronal morphology, revealed by live-cell imaging using lipofection. Circle sizes indicate logarithmically scaled amplitudes of triggered APs, whereas colors indicate the occurrence times of the negative AP peaks relative to the stimulation time. The black arrow points to the stimulation electrode for orthodromic stimulation, whereas the pale red patch indicates the area affected by the stimulation artifact. **(E)** Two axonal branches, labeled “Branch 1” and “Branch 2,” are marked by dark-green and light-green lines on a fluorescence image. White circles indicate the positions of the used recording electrodes. **(F)** AP propagation times obtained from the two branches: average propagation times are presented by solid lines; the standard deviations of the propagation times are represented by the pale bands in the background. Except Panel B, which is an acute cerebellar slice preparation, all other panels refer to cortical neuronal cultures. Images have been adapted with permissions from Bakkum et al., 2013, 2018 **(A–C)**, Radivojevic et al., 2016, 2017 **(D–F)**.

The possibility to stimulate and record from a single axon, simultaneously at multiple spatial locations, enables to study axonal electrical properties in great detail. Capitalizing on this capability, our group investigated the possibilities to study neuronal cultures by using a combination of HD-MEA recordings, electrical extracellular stimulations, live staining of neurons directly on the HD-MEA, and patch-clamping of targeted individual neurons. For a long time, the soma and dendrites have been considered the main contributors to the EAP landscape, since most electrophysiological measurements, e.g., through whole-cell patch-clamp, have been done at the soma. Although the initiation of APs has been known to occur at the distal AIS (Kole et al., 2008; Hu et al., 2009; Foust et al., 2010; Popovic et al., 2011; Baranauskas et al., 2013; Debanne and Garrido, 2018; Leterrier, 2018), the contribution of axons to the EAP landscape has been assumed to be small, if not negligible due to the small dimensions of axonal structures – in contrast to the soma and dendrites. In order to investigate the contribution of different neuronal compartments to the EAP spatial landscape in detail, our group has used HD-MEAs to electrically image EAPs of cultured cortical neurons and of Purkinje cells in acute cerebellar slices (Bakkum et al., 2018). By using spike-triggered averaging, the EAP landscapes of more than 50 neurons were measured and compared to fluorescence images of the respective neuronal cells (Bakkum et al., 2013). We found that the largest measured EAP signal amplitudes originated from the AIS, instead of the soma. The dominant EAP signals, found at the AIS, featured negative polarity (charges entering the cell), while some EAP signals found in nearby dendrites had positive polarity (return currents or charges exiting the cell) (Figure 2A,B). These findings are relevant in interpreting results obtained with extracellular recording schemes (*in vitro* and *in vivo*), for setting up compartmental neuron models, and for developing methods to study the function of the AIS in healthy and diseased cellular cultures.

A characteristic parameter of axons is the conduction velocity, which determines how fast information is transferred between neurons. Detecting fluctuations or deviations in conduction velocity along the axon can provide an understanding of factors that affect conduction success or failure. Such detection poses a major challenge, as it requires a method to directly measure AP propagation at several points along the axon. Several groups have utilized PDMS tunnels, combined with MEAs, to confine the axons, to increase SNR (higher electrical resistance along the channels) and to track the AP propagation along axons and axonal bundles (Shimba et al., 2014; Lewandowska et al., 2015; Habibey et al., 2017). Our group used stimulus-triggered averaging of EAPs to precisely measure the propagation of APs and quantify the conduction velocity along axonal branches (Bakkum et al., 2013). In general, the velocity of AP propagation along axons of wild-type primary rodent neuron cultures increases with age, as observed in our experiments and reported by other authors (Bakkum et al., 2013; Habibey et al., 2017). Both, antidromic (toward the soma) and orthodromic (away from the soma) AP propagations featured variations in conduction velocity along the axon (Figure 2C). The variations persisted upon application of synaptic blockers (100 μM APV, 10 μM CNQX, and 50 μM bicuculline methiodide), suggesting that variations in ion-channel properties and densities influence the conduction properties of axons, among others factors. Moreover, higher conduction velocities were observed in axonal segments closer to the soma as compared to the putatively thinner distal branches, which is in agreement with the theory that the action-potential propagation velocity is inversely proportional to the axon diameter (Goldstein and Rall, 1974). Pathological conditions affecting the axon may cause conduction delays, so that the capability to measure axonal signal propagation may allow for phenotyping cell cultures or brain slices that are characteristic of brain disorders and for identifying pharmacological effects.

The electrical properties of neurons, including their susceptibility to extracellular electrical stimulation, are highly variable across their morphology, so that stimulation efficiency with extracellular electrodes will strongly depend on where the neuron is stimulated. By combining optical imaging and electrically multisite stimulation, we could determine the electrical stimulation profiles of single neurons (Radivojevic et al., 2016). The AIS, the axonal arbor, and proximal somatodendritic compartments could be identified as prime stimulation targets (Figure 2D). Stimulation at the AIS required low voltages and provided immediate, selective and reliable neuronal activation, whereas stimulation at the soma required high voltages and produced delayed and unreliable responses. Subthreshold stimulation at the soma depolarized the somatic membrane potential without eliciting APs. These findings provided a strategy to stimulate individual neurons with high specificity, by first measuring their EAP footprint to determine the likely location of their AIS (region of highest signal amplitudes) for subsequent electrical stimulation with low voltages.

A property of axons, which is of high biological relevance but is very hard to experimentally investigate, is the temporal precision with which axons conduct APs. The dendritic integration depends on the timing of incoming postsynaptic potentials. However, determining the temporal precision of axonal conduction, again, requires experimental access to a single axon at multiple locations and requires resolving single APs. As discussed previously, most recording modalities lack either the spatial or temporal resolution, or they rely on averaging many APs. Averaging, however, is not an option, when the timing of individual APs needs to be estimated. We demonstrated a method to non-invasively and directly record individual APs propagating along axons at microsecond temporal resolution using HD-MEA recordings and a template-matching technique relying on multi-electrode templates. We were able to detect individual APs propagating across entire neurons including axonal terminals, which were hundreds of micrometers away from the AIS by using optimized matched filters (Radivojevic et al., 2017). We found that cortical axons conduct single APs with high temporal precision and reliability. Individual APs travel along 1 mm of axons with a fixed travel time ± 100 μs, and we did not observe any conduction or branch-point failure in more than 8,000,000 recorded APs (Figure 2E,F).

## Discussion and Outlook

Based on the studies reviewed above, we think that HD-MEAs constitute a versatile tool to investigate neuronal information processing and axonal signaling. The possibility to conduct long-term, simultaneous, multisite recordings at high spatiotemporal resolution renders HD-MEAs an ideal technology for detailed characterizations of neurons and axons. HD-MEAs can be used to study alterations in axonal signal propagation and the effects of brain disorders on axonal signaling, as they provide a direct functional readout. Examples include the assessment of the effects of mutations in voltage-gated ion channels (e.g., in the case of channelopathies) on signal propagation velocity. The possibility to reliably electrically stimulate neurons at high frequencies can be used to study the modulation of axonal APs and the mechanisms of axonal conduction failures during repetitive neuronal activation (Geiger and Jonas, 2000; Debanne, 2004; Boudkkazi et al., 2011). Furthermore, spatiotemporal aspects of analog-digital integration in axonal signals can be investigated (Alle and Geiger, 2006; Shu et al., 2006).

HD-MEAs can be arranged in a multi-well-plate format to realize high-throughput as required, e.g., for large-scale genomic interventions (RNAi or CRISPR) or compound screenings, using human iPSC-derived neurons to model neurological diseases. HD-MEAs enable access to a variety of electrophysiological parameters, including axonal properties, which can be used for characterizing functional phenotypes of neurological disease models. Therefore, HD-MEAs can be used as a platform for drug screening, pre-clinical diagnostics and will find applications in the evolving landscape of precision medicine.

From a technological perspective, it is conceivable that only the combination of different recording modalities will substantially increase the number of applications. Extracellular, intracellular and optical readouts can be combined to determine how they can complement each other. For example, subthreshold voltage distributions can be optically visualized while simultaneously measuring axonal signals throughout the axonal arbors using HD-MEAs. Such an approach will allow for deciphering effects of axonal and synaptic plasticity in neuronal networks and provide functional insights into axon physiology and, possibly, pathophysiology. In particular, it may become possible in the future to better understand the functional underpinnings of clinically heterogeneous diseases that arise from axonal disturbances.

## Author Contributions

VE, FF, MO, and AH wrote the manuscript. VE built the figures.

## Conflict of Interest Statement

The authors declare that the research was conducted in the absence of any commercial or financial relationships that could be construed as a potential conflict of interest.
